# The Impact of the Human Genome Project on Complex Disease

**DOI:** 10.3390/genes5030518

**Published:** 2014-07-16

**Authors:** Jessica N. Cooke Bailey, Margaret A. Pericak-Vance, Jonathan L. Haines

**Affiliations:** 1 Department of Epidemiology and Biostatistics, Case Western Reserve University Medical Center, Cleveland, OH 44106, USA; 2 Hussman Institute for Human Genomics, Miller School of Medicine, University of Miami, Miami, FL 33136, USA; E-Mail: mpericak@med.miami.edu

**Keywords:** human genome project, age-related macular degeneration, Alzheimer’s disease, multiple sclerosis, genetics, genomics, genome-wide association study

## Abstract

In the decade that has passed since the initial release of the Human Genome, numerous advancements in science and technology within and beyond genetics and genomics have been encouraged and enhanced by the availability of this vast and remarkable data resource. Progress in understanding three common, complex diseases: age-related macular degeneration (AMD), Alzheimer’s disease (AD), and multiple sclerosis (MS), are three exemplars of the incredible impact on the elucidation of the genetic architecture of disease. The approaches used in these diseases have been successfully applied to numerous other complex diseases. For example, the heritability of AMD was confirmed upon the release of the first genome-wide association study (GWAS) along with confirmatory reports that supported the findings of that state-of-the art method, thus setting the foundation for future GWAS in other heritable diseases. Following this seminal discovery and applying it to other diseases including AD and MS, the genetic knowledge of AD expanded far beyond the well-known *APOE* locus and now includes more than 20 loci. MS genetics saw a similar increase beyond the *HLA* loci and now has more than 100 known risk loci. Ongoing and future efforts will seek to define the remaining heritability of these diseases; the next decade could very well hold the key to attaining this goal.

## 1. Introduction

In celebration of the 10th anniversary of the completion of the Human Genome Project, it is pertinent to take a step back and reflect on the progress that has been made in genetic and genomic research over the past decade by exploring the knowledge gleaned from the extensive wealth of information provided by the Human Genome Project (HGP). Herein we provide a concise historical overview of three signature human diseases that have strong but complex genetic etiologies: age-related macular degeneration (AMD), multiple sclerosis (MS), and Alzheimer’s disease (AD). The significant progress in defining the genetic architecture of these diseases, beginning with the pre-genome-wide association study (GWAS)-era and concluding with the current state of each, and what lies ahead for these complex diseases reflects the great progress that has been made in general in the study of multifactorial diseases, and provides a brief glimpse at what we can hope the next decade of genomic research will provide.

## 2. Age-Related Macular Degeneration (AMD) and the First Genome-Wide Association Study

Age-related macular degeneration an ocular neurodegenerative disease that results primarily in loss of central vision, is a major cause of visual impairment and blindness in elderly populations worldwide. Although there was at one time substantial controversy over the strength of the genetic effects in AMD, genetic and epidemiological research, established that there is a significant genetic component to AMD, estimated to be 45%–70% [1]. This was supported by twin studies that reported higher incidence of disease in monozygotic *versus* dizygotic twins [1,2,3,4,5] and family studies in which risk for developing AMD between first degree relatives ranges from 2–3 [3,6,7]. This knowledge encouraged the application of increasingly sophisticated genomic techniques to elucidate the genetic etiology of AMD susceptibility and pathogenesis. Prior to major genetic breakthroughs such as the completion of the HGP, it was well established that inflammatory and immunologic mediators contribute to AMD (e.g., [8,9,10,11,12,13]). However, this knowledge did not lead to identification of any confirmed genetic loci for AMD. Following the trends at that time in applying the available statistical genetic techniques, numerous genetic linkage studies using multiplex families and (primarily) affected sibships were attempted [14,15,16,17,18,19,20,21,22]. Notably, The *ABCA4* (*ABCR*) locus on chromosome 1p21, identified for its involvement in autosomal recessive Stargardt disease retinopathy [23,24,25,26,27], was one of the first loci identified as involved in AMD, though not all reports have been consistent [23,24,25,26,27,28,29,30,31]. While linkage studies continued to provide suggestive evidence of a role of genetics, they did not find any definitive locus for AMD. In a large meta-analysis of most of these genetic linkage studies, several chromosomal regions were identified as highly likely to harbor AMD genes, most convincingly including chromosome 1q23.3–q32 and 10q26 [32].

With the continuing evolution of HGP resources, in particular the identification of very large numbers of single nucleotide polymorphisms (SNPs) [33,34] multiple new experimental designs for identifying AMD loci were employed. SNPs provided several advantages over the then prevalent microsatellite markers; the two most important were the high density of SNPs across the genome, and their much higher fidelity in genotyping. The culmination of the efforts of four independent studies using four complementary study designs was a convergence on the discovery of the association between AMD and the gene encoding the complement factor H protein (*CFH*), located on chromosome 1q32. In one of the first reported genome-wide association studies (GWAS), Klein *et al.* [35] screened 96 AMD cases and 50 non-AMD controls to evaluate variants associated with AMD. The GWAS method implements a hypothesis-free approach in which a large number of SNPs are genotyped across the genome and evaluated for association with disease. This particular study evaluated 116,204 successfully genotyped SNPs and detected association between AMD and an intronic SNP in *CFH* (*p* < 10^−7^). Linkage disequilibrium analysis and localization using resequencing in this region led to the discovery of a nonsynonymous SNP in exon 9 of *CFH*; this SNP, rs1061170, causes the substitution of a histidine for a tyrosine at amino acid 402 (Y402H).

Independently and concurrently with the Klein *et al.* study [35], Haines *et al.* [36] also identified the association with the Y402H variant, but by implementing a purely locational genomic approach. By focusing on and extending slightly beyond the 24 Mb region implicated by linkage studies of AMD [14,15,22] they identified a five-SNP haplotype spanning a 261 kb region surrounding the Regulators of Complement Activation (RCA) gene cluster by genotyping only 61 SNPs in two independent datasets. Both affected and unaffected individuals homozygous for the risk haplotype were sequenced for the genes residing in this haplotype. Their hypothesis was that having controlled for the locus specific genetic background (e.g., the haplotype), frequency differences for variants between cases and controls would identify the causal variation. Scanning the coding region of *CFH* in those individuals, Y402H was by far the most significantly different of the 11 detected variants. Follow-up genotyping in the original datasets confirmed that the Y402H variant was significantly associated with risk for AMD and that a surprisingly high proportion of the genetic variation in AMD could be attributed to the Y402H variant.

Implementing yet another independent, concurrent, and complimentary approach to localize AMD-causing variants, Edwards *et al.* also identified the Y402H variant using a fine-mapping approach focused on this same general region on chromosome 1 [37]. This study centered efforts on 86 SNPs located in coding sequences encompassing the RCA locus in a case-control sample. The most significant of the 29 associated variants located in the RCA was again rs1061170 (Y402H) in *CFH*. Replication analysis evaluating this and 13 additional SNPs typed in a second case-control sample confirmed the association of Y402H with AMD. Further analysis established that that C (risk)-allele carrying individuals accounted for approximately half of cases.

Hageman and colleagues also confirmed the Y402H variant, applying yet a fourth genetic analysis method [38]. They applied prior biological knowledge of the involvement of *CFH* (also called *HF1*) in membranoproliferative glomerulonephritis type II (MPGNII), a disease in which patients develop ocular drusen nearly identical to those found in AMD patients. The genetic lesion for MPGNII resides in the same chromosome 1q31–32 region that was also implicated in linkage studies of AMD [14,15,22]. Evaluating two samples of unrelated individuals for AMD-associated variation in *CFH*, this group also detected evidence for association between AMD and the Y402H variant.

These four studies simultaneously reported the role of variation in a chromosome 1 region that had previously been highlighted in AMD linkage studies [14,15,22]; identifying this major genetic determinant of AMD, something that even a year earlier was thought not to exist, was a major landmark in genetics of complex disease. These results, while obviously important for AMD research, provided the first validation of the GWAS approach. Up to that point, hundreds of papers had been written about the potential of the GWAS study design, but very little had been published on actual implementation. Dr. Elias Zherhouni, then Director of NIH, highlighted these studies as a major breakthrough in health research [39]. This very strong validation imparted the necessary confidence in GWAS to invigorate its application to numerous other diseases. Over the past nine years more than 2000 GWAS studies that have been published [40].

Since the initial discovery of the Y402H *CFH* variant, substantial progress has been made in understanding the genetics of AMD. This includes the localization of the strongest single genetic effect in AMD on chromosome 10q26 (through positional localization approach) to the region containing *ARMS2* and *HTRA1* (e.g., [41,42,43,44,45,46,47]), though there is still controversy whether either one or both of these genes contains the causal variant (e.g. [46,47,48,49]).

In the *CFH* region, in addition to the high-effect Y402H variant, a deletion of *CFHR1* and *CFHR3* was detected and determine to be protective for AMD [50] (e.g., [51,52,53]). Various additional studies focused on the potential role of additional complement mediators in AMD. Gold *et al.* explored additional alternative complement pathway activators beyond CFH and determined that variants in complement factor b (*BF/CFB*) and *C2* are highly protective against AMD [54]. A coding variant in *C3* was also determined to be associated with AMD [55,56,57]. A variant upstream of the *CFI* gene was also determined to influence AMD risk [58]. A variant in the *CFD* gene was associated with AMD but replicated almost solely in females [59]. More recently discovered AMD-associated loci have been detect in/near genes *ADAMTS9*, *B3GALTL*, *CETP*, *COL8A1-FILIP1L*, *IER3-DDR1*, *LIPC*, *RAD51B*, *SLC16A8*, *TGFBR1* and *TIMP3* [60,61,62].

GWAS studies in AMD now include over 1 million markers [61,63,64,65]. Though the traditional GWAS approach has been incredibly informative for many diseases, a great deal of the genetic proportion of many of these diseases remains to be fully elucidated even after applying straightforward and complex GWAS methods [66]. Approaches to enhance the detection of genetic variation associated with disease have necessarily expanded beyond the traditional GWAS to broaden the range of discovery and increase the power of detection; one technique that aids in this process is imputing variants—using known genetic information from a reference sample. Obviously increasing the sample size of genetic studies of complex diseases is crucial to accelerate the identification of disease-specific variants. Expanding the number of testable variants is now a more attainable goal using imputation, a technique that can significantly increase the number of tested variants beyond those interrogated by a GWAS through informing genotypes of untyped SNPs [67,68]. Combining known genotypes at GWAS-interrogated SNPs with available sequence data from a reference panel and inferring untyped SNPs in the dataset based on haplotype frequencies allows for the inference of numerous SNPs with varying degrees of confidence and accuracy. This method increases the power of GWAS by increasing the number of SNPs that can be tested, it can also lead to more efficient identification of causal variants and/or SNPs in high linkage disequilibrium with a causal variant [67,68]. Imputation has been implemented in several studies of AMD to enhance the ability to detect associated variants [61,63,69]. For example, the most recent publication from the AMD Gene Consortium reports seven novel variants that were detected using imputed data in addition to confirming 12 previously identified variants [61].

An additional method to utilize genome-wide data beyond the traditional association analysis is to perform pathway enrichment analyses. The goal of such analyses is identify biological relationships between associated genetic signals and pathways of interest in a particular disease. Pathway analysis can be performed by a comprehensive review of GWAS results to assess overrepresentation of SNPs meeting a specific threshold that occur within biological pathways [70]. These enhance GWAS by evaluating potentially biologically relevant signals that might otherwise be overlooked because of the numerous false-positive results that occur in large GWAS studies [70]. These have the potential to highlight otherwise undetected small and/or interactive effects that are important to evaluate in addition to and in the context of the overall genome-wide results. Using the INRICH (Interval-based Enrichment Analysis Tool for Genome Wide Association Studies) pathway analysis tool [71] to evaluate overall results, the AMD Gene Consortium not only confirmed previously implicated AMD pathways, but also determined additional pathways of interest in the most recent publication which detected enrichment of complement and atherosclerotic pathway-encoding genes as well as genes involved in pathways of collagen and extracellular region, complement and coagulation cascades, lipoprotein metabolism, and regulation of apoptosis [61].

The impressive impact that genetic information can have on our understanding of disease pathophysiology is highlighted in the recent publication by Yang *et al.* in which they report that *ARMS2/HTRA1* risk alleles contribute to AMD pathogenesis by decreasing the defense capabilities of superoxide dismutase 2 (SOD2) and thereby cause the retinal pigment epithelium to be more susceptibility to oxidative damage [72]. Having an explanation for the role the variants have in the disease is crucial to further elucidating disease mechanisms both genetically and physiologically. Additionally, genetic studies implicate VEGF as having a role in AMD and current AMD treatment and clinical trials utilize this information for treatment of neovascular AMD (reviewed in [73]), thus highlighting the utility of genetic data for clinical impact.

## 3. Alzheimer’s Disease

Alzheimer’s disease (AD) is a genetically heterogeneous neurologic disorder that is the leading cause of dementia among the elderly. It is characterized by the progressive loss of cognitive ability beyond what is normally associated with aging. AD is a complex disease that is influenced by both environmental and genetic mediators, the most significant of which is age [74,75]. The heritability of AD is estimated between 60%–80%. Before 1985, there was very significant debate about whether or not genetics played any role in AD (e.g., [76,77,78,79,80]). However, in 1987, using some of the earliest technologies employing genomic markers, a locus for the rare early onset AD (EOAD) was identified [81], and in 1991 the responsible variation in the *APP* gene was located [41].

Expansions of genomic marker sets, developed through early HGP efforts, were used to further identify two additional early onset genes in the early 1990’s [82,83,84,85,86]. Simultaneously and independently, the emerging technologies of genomic markers and genetic linkage analysis were applied to the far more common late onset Alzheimer’s disease (LOAD), which accounts for 99% of AD cases [87]. Using these techniques, Pericak-Vance *et al.* identified a locus on chromosome 19 near the gene encoding apolipoprotein E (*APOE*) [88,89], which was at that time thought to only be involved in cardiovascular disease. This locus has three distinct alleles: ∈2, ∈3, and ∈4. Corder *et al.* characterized a dose-dependent association between the *APOE*-∈4 allele and an increased risk of LOAD [90]. Mutations in the EOAD genes are causal, with very high penetrance, and opened avenues for exploring the pathophysiology of AD. However, in aggregate they explain less than 1% of AD. In contrast, *APOE* explains at least 25% of AD. A year after determining the role of the ∈4 variant in LOAD susceptibility, it became apparent that the ∈2 allele provided an independently protective effect on LOAD [91].

The *APOE* finding was pivotal for two reasons. Within the AD research community, it provided a new avenue and a completely different view of the genetic etiology of AD. More generally, however, this was one of the very first examples of how the emerging technologies of the HGP could be successfully applied to diseases lacking a simple Mendelian inheritance pattern, *i.e.*, what are commonly called complex diseases. The finding of the *APOE*-∈2 allele protective effect was also one of the first examples of different alleles carrying different effects on a complex disease, a pivotal moment in AD research and broadly in the field of genetics.

Innumerable attempts to identify additional genomic variations modulating the risk of LOAD followed these groundbreaking *APOE* discoveries, using the increasingly dense set of known variations and emerging sequencing techniques (cataloged in Alzgene.org). These efforts were primarily applied to specific genes of interest; that is, employing a focused candidate gene approach. Although there were numerous reports of significant associations, no consensus arose that any of these were true effects. It was not until GWAS became a viable approach [92,93,94], and multiple datasets were combined, that additional LOAD loci become visible and confirmed [95,96,97]. The most recent efforts by the Alzheimer Disease Genetics Consortium (ADGC) and the International Genomics of Alzheimer’s Project (IGAP) have greatly increased the number of known loci associated with LOAD. In the 2011 Naj *et al.* report, a three-stage design (discovery stage 1, replication stages 2–3) was utilized; this analysis evaluated >18,000 cases and >29,000 controls using both joint- and meta-analysis approaches and novel genome-wide significant hits were detected at SNPs in *MS4A4A*, *CD2AP*, *EPHA1* and *CD33* [96]. In Lambert *et al.* 2013, the IGAP reported an additional eleven novel LOAD susceptibility loci after analyzing genotyped and imputed data in a two-stage meta-analysis of >25,000 cases and >48,000 controls [95]. There are now over 20 loci identified that influence LOAD [95]. Importantly, using the pathway approach, the amyloid precursor protein and tau pathways are confirmed by this most recent large GWAS in addition to the newly implicated hippocampal synaptic function, cytoskeletal function and axonal transport, regulation of gene expression and post-translational modification of proteins, and microglial and myeloid cell function pathways [95].

## 4. Multiple Sclerosis

Multiple sclerosis (MS) is a common cause of neurological disability involving inflammatory demyelination of the central nervous system [98,99,100,101]. There is ample evidence that MS has a strong genetic component, but like so many other complex diseases, non-genetic influences are also important (e.g., [99,102,103,104]). MS is also a complex, heterogeneous disease in which significant efforts to unravel the role of genetics have been made. Unlike both AMD and LOAD, the first and strongest genetic effect in MS was identified well before the HGP was undertaken. Because MS is an autoimmune disease, it was strongly suspected that the major histocompatibility locus (*MHC*) would be involved. More specifically, there was a focus on the human leukocyte antigen loci on chromosome 6. In the early 1970’s the *HLA* loci could be genotyped using blood antigen reactions, allowing assignment of genotypes without directly examining the DNA. Through a number of efforts (e.g., [99,103,104,105,106,107,108,109]) a strong risk association with the *HLA-DR* locus, and specifically the 15*01 allele was identified.

Despite this auspicious beginning, identifying additional MS loci languished. As with the other complex diseases, genetic linkage analysis was applied to multiplex MS families, with varying results. Some early genetic linkage studies confirmed the role of HLA [110], while others did not [111]. Additional studies, using the increasingly dense DNA marker sets and larger datasets, ultimately demonstrated and confirmed that the *HLA* locus was the single largest genetic effect, and that any other MS loci would have at most modest individual effects [109,110,112,113,114,115,116]. These studies did highlight several other possible loci, but did not have the resolution to identify specific associated genetic variations [115,116].

Finally, in 2007, nearly 30 years after the initial association finding, a second locus for MS was identified [117,118]. Gregory *et al.* employed a genomic convergence approach that integrated data from genetic linkage studies, genetic association studies, model system gene expression data, and *in vitro* functional data to narrow in on a specific locus and a functional polymorphism in the interleukin-7 receptor α chain (*IL7R*) [117]. Independently, the International Multiple Sclerosis Genetics Consortium (IMSGC) published results from one of the first large-scale GWAS studies, using 334,923 GWAS SNPs. The IMSGC used a hybrid study design that included a family-based study of 931 family trios and an independent dataset set of cases and controls [118]. These analyses confirmed the role of genetic variation in *IL7RA* and also highlighted variations in *IL2RA*.

These results also had broad implications for the field of MS resarch. The IMSGC GWAS was still one of the first such studies done with a well-powered dataset and demonstrated that family-based and case-control GWAS approaches were both useful methods for exploring genetic information. In addition, like AMD, the convergence of independent approaches (GWAS and gene-targeted methods) further validated that GWAS could identify relevant associated loci. Subsequent studies with much larger datasets [96,119,120] have now identified over 100 total loci associated with MS.

Efforts in MS have shown substantial increases in the number of independent loci associated with this disease. The most recent IMSGC study evaluated in two stages more than 80,000 individuals of European ancestry [119]. This analysis expanded the known MS loci by 48, raising the total number of discrete MS-associated loci to 103. In addition, the IMSGC interrogated specific genomic regions and hypotheses using a custom array, the Immunochip. The group efficiently utilized extensive amounts of data by assembling multiple studies and utilizing imputation methods and then applying conditional and joint analysis methods [119]. Such methods are becoming more common in the efforts to expand the power to detect common variation in many multifactorial diseases. The strongest of the novel hits from this analysis implicates a SNP in the region between *BCL10* and *DDAH1* [119]; *BCL10* is an activator of nuclear factor (NF)-κB signaling which is involved in gene expression control of inflammation, immunity, cell proliferation and apoptosis and has been explored as a clinical target for MS [73,119,121].

Pathway analysis in MS has also proven useful. The IMSGC study additionally sought to evaluate the Gene Ontology (GO) processes of the associated variants using the MetaCore ([122]); their results indicated, as expected, that most variants fall in or near genes with immune function [119]. Another recent endeavor to evaluate pathways involved in MS utilized results from eight MS GWAS datasets and prioritized genes in the cell adhesion molecule (CAM) biological pathway with the Cytoscape software [120,123]. Their findings highlighted five networks that were associated with susceptibility to MS—again supporting the utility of expanding beyond traditional case-control association analyses of GWAS data and encouraging the use of multiple datasets to determine enrichment of signals that might otherwise not have been detected using a traditional GWAS approach [120].

## 5. Conclusions and Future Directions

Since the completion of the HGP and shortly after the first GWAS, thousands more GWAS have been reported [40]; these have brought forth great progress in numerous diseases that previously were only hypothesized to have a genetic component. Large-scale collaborative efforts have raised the number of known AMD, AD, and MS loci to 19 [61], 20 [95], and 103 [119], respectively. Efforts to increase sample size have been successful, as evidenced by the largest and most recently reported analyses of AMD [61], AD [95] and MS [92], which each evaluated >74,000 individuals; however, other techniques are necessary to evaluate and explore as data becomes increasingly large and complex. Whole exome and whole genome sequencing are more recent approaches to generating genetic data that allow investigation far beyond the capabilities of the GWAS, and their utility is just starting to take shape in studies of many complex common diseases, including those mentioned herein. These will enable the study of rare and low frequency variants, which have been implicated as a potential source of missing heritability in many genetic diseases [66]. Analysis of data from exome arrays, designed to jointly interrogate data relevant to association studies of common variants and sequencing studies of rare variants, will improve genetic analysis of disease by providing greater coverage of known susceptibility loci and enhancing the likelihood for discovery of novel disease loci.

For each of these diseases, the fact that there is a genetic component is irrefutable; this knowledge has, over the past decade, most certainly been confirmed and expounded upon with the completion of the human genome sequence. As genetic knowledge continues to grow, and as clinical phenotyping techniques improve, further genetic variation influencing AMD, AD, and MS will likely be detectable and, hopefully, their roles in these diseases will be more clearly defined [124]. We can anticipate that as our understanding of genetic etiology of these diseases grows, future studies will further explore rare variations contributing to disease, the role of copy number variants, and the genetics of these diseases in non-European populations. Additionally, the role of currently undetermined environmental factors and their interactions with genetic variants must continue to be elucidated. The global objective of prior and ongoing studies is certainly to improve the current comprehension of new and existing disease loci in order that the biology of these diseases can be fully explicated in the hope of attaining improved strategies for disease treatment and prevention in the future.

The last decade has doubtlessly ushered in dramatic advances in the amount of shared data available to genetic researchers. Resources such as the NHLBI Grand Opportunity Exome Sequencing Project [125], the 1000Genomes [126], ENCODE [127], and the International HapMap Project [128,129] provide seemingly limitless amounts of data—all geared toward further understanding the intricacies of the human genome and how alterations of it influence human variation. We have provided a brief genetic history of three diseases that are exemplars of developing approaches to apply the incredible resources of the HGP. Such progress will most certainly continue to improve and exponentially increase in the next decade to facilitate a greater understanding of these and other complex diseases, as well as usher in the realization of personalized medicine.
